# Associations between fasciolosis and milk production, and the impact of anthelmintic treatment in dairy herds

**DOI:** 10.1007/s00436-017-5481-3

**Published:** 2017-06-06

**Authors:** Kerstin Köstenberger, Alexander Tichy, Karl Bauer, Peter Pless, Thomas Wittek

**Affiliations:** 1 University Clinic for Ruminants, University for Veterinary Medicine Vienna, Veterinärplatz 1, 1210 Vienna, Austria; 2 Department for Biomedical Sciences, University for Veterinary Medicine Vienna, Veterinärplatz 1, 1210 Vienna, Austria; 3 Styrian Animal Health Service (AHS), Graz, Austria; 4 Department of Veterinary Administration, Styrian Government, Graz, Austria

**Keywords:** *Fasciola hepatica*, Dairy cattle, ELISA, Milk production, Risk factors, Treatment

## Abstract

Liver fluke is a ubiquitous parasite that causes extensive production losses in cattle and is a zoonosis. The aims of this study were to determine the prevalence of fasciolosis in 178 dairy cattle herds in Styria (federal state of Austria) and its influence on production, to detect the risk factors for infection, and to explore effective strategies in management and control. A questionnaire on farm management, prophylaxis, and therapy was developed and applied. Furthermore, production parameters (milk yield, milk protein content, butter fat content, non-return rate 90, calving to conception interval, service period) were recorded for 2014 and 2015, and a commercial ELISA for detection of *Fasciola hepatica* antibodies was applied in bulk tank milk in March 2014 and March 2015. Analysis of bulk tank milk samples showed a prevalence of 61.3% in 2014 and 45.5% in 2015. No associations could be found between *F. hepatica* exposure and farm structure or pasture management. Farms with highly positive (optical density ratio (ODR) ≥ 0.6 and lying above the upper interquartile range) antibody levels had a significantly lower annual milk yield of 438 kg per cow per year (*p* = 0.045), butterfat content of 0.091% (*p* = 0.004), and milk protein content of 0.046% (*p* = 0.024). However, fertility parameters were not significantly associated with liver fluke exposure. Anthelmintic treatment led to significantly lower antibody levels in the subsequent year (*p* = 0.042) and had a significant influence on protein content in milk (*p* = 0.003). This study highlighted the importance of fasciolosis in Austria and its influence on milk production and the need for veterinary advice regarding prophylactic measures to reduce economic losses.

## Introduction

*Fasciola hepatica* is a ubiquitous parasite of cattle and sheep being found in all inhabited continents. In Europe, prevalence based on herd-level antibodies range between countries from 7% in south central Sweden (Höglund et al. 2010), 18% in Switzerland (Rapsch et al. 2006), and 23.6% in Germany (Kuerpick et al. 2013) to 78% in Ireland (Selemetas et al. 2015a) and 79.7% in Great Britain (Howell et al. 2015). For Austria, Matt et al. 2007 could show a prevalence of 73% in the federal state Tyrol. In Carinthia (federal state of Austria), a prevalence of more than 90% was reported in individual serological tests on farm level (Duscher et al. 2011).

Weather and pasture conditions are the major influences on the incidence of liver fluke infections as these control the habitat and so the population of the recognized intermediate host, the snail *Galba truncatula*. High rainfall intensity at the appropriate time, coupled with poorly drained loamy soils leading to oligotrophic standing waters, is risk factor for the presence of the intermediate host and therefore for the infection with *F. hepatica* (Bennema et al. 2011; Charlier et al. 2011; Howell et al. 2015; Selemetas et al. 2015b). Other parameters such as drinking water systems on pastures (Selemetas et al. 2015a; Charlier et al. 2011), herd sizes (Charlier et al. 2011; Selemetas et al. 2015a; Howell et al. 2015), and grazing season length (Bennema et al. 2011; Selemetas et al. 2015a) have been described in literature as further risk factors all leading to an increase of oligotrophic troughs where the snails can breed thus allowing rapid multiplication of the parasite.

In many cases, fasciolosis is a subclinical disease presenting with herd- or flock-level production losses rather than the classic clinical signs in individuals. In Switzerland, the nationwide costs of infection with liver fluke were estimated at €52 million, or €299 per infected animal per year (Schweizer et al. 2005).

Infection with *F. hepatica* results in decreased milk yield (Charlier et al. 2007; Khan et al. 2009; Mezo et al. 2011, Charlier et al. 2012; Howell et al. 2015). Some studies reported a decrease in butter fat content (Charlier et al. 2007; Khan et al. 2009) while others could find no evidence of this (Mezo et al. 2011; Charlier et al. 2012; Howell et al. 2015). Similarly, while some describe effects on fertility such as increased inter-calving interval (Charlier et al. 2007), decreased conception rate (Oakley et al. 1979), and delay in the onset of puberty (Lopez-Diaz et al. 1998), others have not observed any differences in the reproduction parameters they studied (Mezo et al. 2011; Howell et al. 2015).

Fasciolosis has also negative effects on carcass quality at slaughter. Sanchez-Vazquez and Lewis (2013) reported lower weight and lower fat levels of carcasses from animals with liver fluke infestation. Further liver condemnation of infected animals leads to substantial visible economic losses (Schweizer et al. 2005; Radfar et al. 2015).

Vercruysse and Claerebout (2001) proposed a herd prevalence of fasciolosis equal or over 25% as a threshold for significant production losses. Strategies to prevent and treat fasciolosis have been developed to minimize these losses. Primarily, snail control must be guaranteed. Pasture management like fencing and draining of wet soils or the pasture rotation system to decrease the infection risk has been described by Boray (1971). In addition, anthelmintic treatment to reduce parasitism in the host and therefore the egg elimination and pasture contamination has been propounded (Roberts and Suhardono 1996; Boray 1971; Torgerson and Claxton 1999; Knubben-Schweizer et al. 2010). However, long milk and meat withdrawal times of most therapeutic agent treatment and prophylactic options are typically limited in dairy cows suffering from fasciolosis (Khan et al. 2009; Charlier et al. 2012).

The aims of this study were to assess the occurrence and importance of *F. hepatica* in dairy herds in Styria and to detect the risk factors for fasciolosis and its influences on production. Furthermore, it was an aim to explore which control strategies are suitable for dairy herds of this region. In Austria, approximately a quarter of the 2 million cattle were dairy cows kept on 61.000 farms with an average of 32 cattle per herd in 2015 (Statistik Austria 2015). Alpine pasture and smallholder farms are typical for Styria (federal state of Austria).

It has been hypothesized that farm management, prophylaxis, and treatment are associated with production and health parameters. Pasture management and anthelmintic treatment are supposed to lower the infection risk and lead to healthy high-performance herds.

## Materials and methods

### Study design and population

The study population consisted of a convenient sample of 178 dairy herds (1.7% of Styrian cattle herds, Statistik Steiermark 2015) and a total number of 3.934 dairy cows (3% of the amount of Styrian cattle and 4.8% of the Styrian dairy cow population, Statistik Steiermark 2015) with an average number of 22 dairy cows per herd. These herds were located in a district of the upper region of Styria typical of alpine farming and grazing practices. Criteria for participation were the membership in the Styrian Animal Health Service (Verein Steirischer Tiergesundheitsdienst (TGD) Graz, Austria) and in the Milk Recording Service Styria (Landeskontrollverband (LKV) Steiermark, Austria). All potentially suitable dairy herds in the area had been asked to participate by the LKV; the 178 herds joined the study voluntarily. The farmers of the herds were invited to participate in a questionnaire and gave their consent for using their milk test results and herd information regarding their production parameters for the purpose of research.

### Questionnaire

The questionnaire was developed in 2015 and consisted of four parts: farm structure (A), grazing management (B), pasture hygiene management (C), and anthelmintic treatment (D) similar as recommended by Selemetas et al. (2015a). The 23 questions included 5 open-answer questions, 12 multiple-choice questions, and 6 binary questions. The questionnaire was conducted face to face by the corresponding author.

Section A (farm structure) determined the total amount of cattle; the number of dairy cows and young stock; the predominant breed kept on the farm; if sheep, goats, and horses shared pasture with the cattle; and if there were deer or other wild ruminants seen on the pasture.

Section B (grazing management) asked about the total area of grassland (ha), the number of paddocks, and if the cattle grazed the whole year day and night, and the whole year during the day or grazed seasonally (length in months). Furthermore, it was asked how the pasture was used (ration grazing, rotation pasture, continuous grazing, or alpine pasture) for dairy and for young stock. Farmers were asked about the quality of pasture drainage (good, moderate, poor), the percentage of grassland (<10, <25, 25–50, 50–75, >75%) with snail habitats (streams, ponds, flooded ditches), the way drinking water was provided on the pasture (drinking bowl, trough, pond, burn, river), and if they had changed anything in their grazing management since they were informed about the antibody levels against *F. hepatica* of their farms (drainage, fencing, rotational pasture management).

Section C (pasture hygiene management) asked farmers about general hygiene management in the herd and if they had changed anything regarding the pasture hygiene since they were informed about the antibody levels against *F. hepatica* of their farms (e.g., not using silage or storing hay for a couple of months if the grass was harvested from moist pastures).

Section D (anthelmintic treatment) asked about treatment in general, the drug used, the dosage, if all cattle were treated and if not which were treated, and the frequency and time of use.

### Detection of *F. hepatica* exposure

Bulk milk tank (BMT) samples were supplied by the Styrian Animal Health Service. After collection, BMT samples were stored for no longer than 7 days at between 2 and 4 °C. The Veterinary Laboratory of TGD tested bulk tank milk samples in March 2014 and March 2015 using SANOVIR® *F. hepatica*-Ab (Boehringer Ingelheim Svanova, Sweden), a commercial *F. hepatica* excretory-secretory (ES) antigen ELISA, according to Charlier et al. 2007, which is a modification of Salimi-Bejestani et al. (2005b). Antibody levels were expressed as an optical density ratio (ODR) with the formula ODR = (OD − NC) / (PC − NC), with OD as the optical density of the sample and NC and PC as the OD of the negative and positive controls (Charlier et al. 2007). The sensitivity and specificity of this Ag-detecting ELISA to detect herds in which more than 25% of the cows are infected are 96% (95% CI 89–100%) and 80% (95% CI 66–94%), respectively (Salimi-Bejestani et al. 2005a).

### Production parameters

Milk parameters (milk yield, milk protein content, butter fat content) and fertility variables (non-return rate 90 (ratio of cows, which not get seeded again within 90 days), calving to conception interval, service period) were provided by the Milk Recording Service Styria, Austria, which is routinely recording monthly milk and fertility parameters. Data were available for all herds participating in the study during the periods October 2013–September 2014 and October 2014–September 2015.

### Statistical analysis

Data were stored in Excel and imported into SPSS Statistics version 20.0. (IBM Corp., USA) for statistical analysis. Standard distribution was tested with Kolmogorov-Smirnov test. For a detailed overview, farms were divided into three groups (Charlier et al. 2012): highly positive herds or high (H) having an ODR ≥ 0.6 and lying above the upper interquartile range, herds having an ODR between 0.6 and 0.3 defined as slightly positive or medium (M), and herds with an ODR < 0.3 as “negative” or low (L).

For further statistical analysis, slightly positive and negative herds were summarized in one group.

To compare the parameters, a *t* test for independent samples was used. Influences of treatment on ODR and production parameters were calculated by variance analysis. Associations between variables were assessed calculating Pearson correlation coefficients. Results were considered significant at *p* < 0.05.

## Results

### Prevalence of *F. hepatica*

During the 2 years of the study, a general decrease of ODR (ODR 2014 = 0.408, median 0.372, IQR 0.34; ODR 2015 = 0.341, median 0.274, IQR 0.32; *p* < 0.001) was observed. In 2014, the overall total herd prevalence of fluke infection was 61.3% (109 of 178 herds) which could be subdivided in 22.5% (40 herds) highly positive and 38.8% (69 herds) slightly positive herds. In 2015, the overall herd prevalence was 45.5% (81 of 178 herds) divided in 16.3% (29 herds) highly positive and 29.2% (52 herds) slightly positive herds.

### Farm structure and pasture management as risk factors

The questionnaire resulted in the following results: on average, the farms had 53 cattle in total, (22 dairy cattle and 30 young stock), 4 set pastures or paddocks, and an overall pasture area of 16 ha. Detailed information is shown in Table 1.

**Table 1:** Descriptive statistics of farm structure parameters (*n* = 178)

Variable	Minimum	Maximum	Mean	SD
Total cattle (*n*)	10	400	53	40
Dairy cattle(*n*)	5	80	22	13
Young stock (*n*)	0	320	29	28
Pastures/paddocks (*n*)	0	14	4	2
Pasture size (ha)	0	110	16	14

Simmental was the predominant breed with 92.1%. Only 6.2% of all the farms kept other animals than cattle on their pastures. In 88.8%, paddocks were used seasonally (rarely or not during winter). The season lasted around 6 months on average ($\overline{\mathrm{x}}\;6.192$; σ 1.319) depending on the geographical location of the farm. The remaining results are shown in Table 2.

**Table 2:** Summary of variables concerning pasture management (*n* = 178)

Categorical variables	% of farms positive for variable
Grazing management	
Ration grazing	46.60
Rotation pasture	48.9
Continuous grazing	24.7
Alpine farming dairy cattle	18.5
Alpine farming young cattle	71.9
Drainage of pastures	
Good drainage	72.2
Moderate drainage	26.7
Poor drainage	1.1
Supply of water on pastures	
Drinking bowl	38.9
Trough	60.6
Pond, stream, river	52.6

None of the variables on farm structure or pasture management were significantly correlating with ODR values.

### Influence on milk production and fertility

Parameters describing milk production were negatively correlated with ODR values: milk yield (kg) (*r* = −0.211, *p* < 0.001), butterfat (%) (*r* = −0.235, *p* < 0.001), and milk protein (%) (*r* = −0.256, *p* < 0.001). Highly positive herds showing an ODR over the interquartile range had significantly lower milk yield, butterfat content, and milk protein content. Compared to slightly positive or negative farms, there was a decrease in annual milk yield of 438 kg, or 6% (measured on the mean milk yield) per cow per day (*p* = 0.045). Furthermore, a decrease in butterfat of 0.091% (*p* = 0.004) and in milk protein of 0.046% (*p* = 0.024) in highly positive herds was found. Service period, calving to conception interval, and the 90% non-return rate did not show any significant changes. A summary of the data is shown in Table 3.

**Table 3:** Influence of highly positive ODR values (≥0.6) on production parameters; highly positive farms (*n* = 40), slightly positive/negative farms (*n* = 138). *NRR90* non-return rate 90, *CCI* calving to conception interval, *SP* service period

Variable	Highly positive farms mean	Slightly positive/negative farms mean	*t*	*p* value	Mean difference
Milk yield (kg/year)	6981.4	7419.412	2.009	0.045	438.012
Butter fat (%)	4.1	4.192	2.919	0.004	0.091
Protein (%)	3.403	3.449	2.266	0.024	0.046
NRR90	65.23	63.883	−0.602	0.548	−1.347
CCI	396.037	390.01	−1.291	0.198	−6.027
SP	107.027	100.197	−1.539	0.125	−6.83

### Effect of anthelmintic treatment

A total of 50 farmers (28%) treated their herds with an anthelmintic drug supposedly effective against trematodes after they had been informed about their herds having high ODR values. Most of these herds (93.8%) were treated with a combination of closantel and ivermectin pour on (Closamectin®, Norbrook Laboratories Limited, Great Britain). All farmers stated that they applied 10 ml/100 kg body weight, as recommended by the manufacturer. The second drug used was albendazol (Valbazen®, Zoetis, Austria, Albendazol-ani-medica®, Animedica, Germany), applied as oral suspension 4 ml/10 kg body weight (Valbazen®) or 10 ml/100 kg body weight (Albendazol-ani-medica®). Dairy cattle were treated at drying off whereas young stock was treated before and/or after grazing on pasture. The treatment of herds which had ODR values ≥0.6 resulted in a significant decrease of ODR values (ODR 2014 = 0.698; ODR 2015 = 0.508; *F*(1,38) = 4.41; *p* = 0.042). There was no significant effect of treatment of herds with ODR values <0.6 (*F*(1136) < 1; *p* = 0.768). Detailed information is shown in Table 4. Treatment showed also a significant influence on protein content in the milk. Content of protein could be held on a significantly higher level if herds were treated where necessary (ODR ≥ 0.6), whereas it was constantly decreasing in herds that were not treated (*F*(1,38) = 10.092; *p* = 0.003). However, treatment did not result in significant influence on milk yield (*F*(1,38) < 1; *p* = 0.437) and butterfat content (*F*(1,8) < 1; *p* = 0.657) of the subsequent year. Detailed information is provided in Tables 5 and 6.

**Table 4:** Influence of treatment on ODR values (*n* = 178)

		ODR 2014			ODR 2015			Percentage of farms with decrease in ODR (%)
	*n*	Median	25% percentile	75% percentile	Median	25% percentile	75% percentile	
Treated								
ODR ≥0.6	23	0.689	0.666	0.749	0.528	0.408	0.618	95.7
ODR 0.3–0.6	21	0.498	0.404	0.548	0.403	0.315	0.510	57.1
ODR <0.3	6	0.222	0.170	0.270	0.121	0.083	0.183	66.7
Not treated								
ODR ≥0.6	17	0.696	0.634	0.752	0.643	0.525	0.833	58.8
ODR 0.3–0.6	48	0.411	0.352	0.485	0.288	0.202	0.435	77.1
ODR <0.3	63	0.200	0.161	0.260	0.182	0.125	0.241	63.5

**Table 5:** Influence of treatment on production parameters, farms with ODR ≥0.6 and treated *n* = 23

	Variable	Mean	Minimum	Maximum	SD
Before treatment (2014)	ODR	0.698	0.6	0.808	0.054
	Milk yield (kg)	7172.167	4102.204	11,082.08	1859.365
	Protein (%)	3.422	3.14	3.65	0.16
	Butter fat (%)	4.136	3.57	4.6	0.243
After treatment (2015)	ODR	0.508	0.121	0.775	0.158
	Milk yield (kg)	7112.212	4019.496	12,302.436	1957.33
	Protein (%)	3.408	3.13	3.63	0.142
	Butter fat (%)	4.079	3.59	4.58	0.259

**Table 6:** Influence of treatment on protein content (%) in milk, farms with ODR ≥0.6, farms treated *n* = 23, farms without treatment *n* = 17

Variable	Mean	Minimum	Maximum	SD
Protein (%) with treatment				
2014	3.422	3.14	3.65	0.16
2015	3.408	3.13	3.63	0.142
Protein (%) without treatment				
2014	3.401	3.15	3.62	0.131
2015	3.303	3.08	3.6	0.142

## Discussion

The ELISA testing of the BTM samples showed an exposure of the dairy herds of 61.3% in 2014 and of 45.5% in 2015 based on the H (high) and M (medium) criteria (as explained in “Material and methods” section). Comparisons with other studies are difficult to draw since cut-off levels differ between ELISA results. Duscher et al. (2011) showed a prevalence of 60%, found by bulk milk ELISA on herd level over more than 1 year in Carinthia (federal state of Austria). These results differ from those found in Ireland (78%) by Selemetas et al. (2015a) and in Great Britain (79.9%) by Howell et al. (2015). The reason may either be the different environmental and climatic conditions or that the samples in the present study were collected in March and the others in October to December (Howell et al. 2015; Selemetas et al. 2015a). Since due to pasture contamination the peak of the annual life cycle of *F. hepatica* is expected at the end of the summer (Salimi-Bejestani et al. 2005a), the antibody levels would be higher in autumn.

No influence of small ruminants, horses, or deer sharing pastures with cattle on antibody levels was found although they are potential hosts for liver fluke. However, the percentage of farmers keeping horses and small ruminants together with cattle in this study was very low (6.2%) so the importance of these species remains open. Alpine farming in Austria leads to high incidence of deer on the pastures. In this study, nearly 90% of all farmers confirmed deer sightings on their pastures. Therefore, it was not possible to assess the influence of wildlife on bovine fasciolosis.

Although the impact of poorly drained, loamy pasture, high rainfall, and flooded parts of the soil on the incidence of fasciolosis is known (Bennema et al. 2011; Charlier et al. 2011; Howell et al. 2015), the current study could not show higher risk of farms on which the farmers stated that they have poorly drained or boggy pastures. The explanation could be that some farmers may have wrongly assessed the status of their pasture or were unwilling to give the correct answer. Only 1.1% of the farmers confirmed having poor drainage status on their pastures, whereas 72.2% valued their pastures having a good drainage status.

The present study showed a reduction of milk yield of around 6% (438 kg/cow/year) between H and M/L herds. Several other studies reported an association between liver fluke infection and losses in milk yield with similar results (Charlier et al. 2007; Khan et al. 2009; Mezo et al. 2011; Charlier et al. 2012). However Howell et al. (2015) showed a reduction of 15% in milk yield (based on 32 herds with similar results in 475 herds where milk yield was not measured but estimated), probably because of the high prevalence found in this study.

In 2015, the average milk price was €0.34 per kg (Agrar Markt Austria 2015); thus, a reduction in annual milk yield of 438 kg results in a financial loss of €147.96 per cow per year, resulting in a loss of €3255.12 on herd level (measured on the mean dairy herd size of this study of 22 cows). Possible additional losses of body condition are not considered as this was not measured.

Fluke infection had a significant effect on the content of butterfat and milk protein in the current study. Some previous studies (Charlier et al. 2007; Khan et al. 2009) found similar associations for butterfat, but protein content effects were inconsistent (Charlier et al. 2007; Mezo et al. 2011; Charlier et al. 2012; Howell et al. 2015). Changes in these parameters might be more obvious in the current study, since the predominant study breed was Simmental, a breed with naturally high milk butterfat and protein. Like Mezo et al. (2011) and Howell et al. (2015), no associations between high antibody levels and fertility parameters were found, although others have found an effect (Oakley et al. 1979; Lopez-Diaz et al. 1998; Charlier et al. 2007).

Treatment showed effect on antibody levels and on milk protein in highly positive herd content in the current study. There were no influences on milk yield and butterfat. Former studies reported a decrease of ODR values (Charlier et al. 2012) and increases of milk yield (Khan et al. 2009; Charlier et al. 2012) and butterfat (Khan et al. 2009) after treatment on individual cow level. Comparison is limited with Charlier et al. (2012) since this study only found changes of milk yield in herds with ODR of 0.3–0.5 and not ≥0.5. The current study worked with values of ODR ≥ 0.6.

Overall, this study showed that fasciolosis does have an impact on production and that treatment can lower the burden. Comparison between the financial loss of well over €100 per cow/year in H herds and the treatment costs of around €35 per cow/year indicates the value of such treatment not only for the economic reason but also for the healthiness of the herds. We suggest the same follows for M herds. It has to be considered that this study was only over 1 year and the long-term effects of such targeted annual treatments in lowering challenge may well be cumulative.
